# 900. Let’s Win! Scrub In!

**DOI:** 10.1093/jbcr/irag033.396

**Published:** 2026-04-06

**Authors:** Lazon Coleman

**Affiliations:** Jacobi Burn Center, Brooklyn, NY

## Abstract

**Introduction:**

It is not a standardized practice to “scrub in” on a critical care unit. It is believed that the number of Central Line-Associated Bloodstream Infections (CLABSIs) in the Medical Intensive Care Unit (MICU) are higher from the burn unit at our institution due to our difference in current practice of hand hygiene. Upon our arrival to the burn unit we scrub in using an antimicrobial solution like chlorhexidine or povidone iodine with a sterile brush or an alcohol-based rub. This practice removes bacteria and other microorganisms from our hands. In burn units, preventing CLABSIs are important and critical due to the high risk of infection in burn patients. Preventing infection in a burn unit requires strict adherence to infection control protocols, such as proper scrubbing, using sterile techniques, and environmental disinfection. Burn patients are vulnerable to infections due to the loss of their natural skin barrier. Hand hygiene is a key component in preventing infection. The purpose of this study is to compare the burn unit’s practice to the MICU’s practice within our institution to show that this technique of hand hygiene is not only effective in our burn unit but can potentially decrease the infectious burden in other intensive care units.

**Methods:**

The Quality Assurance department tracks the number of CLABSI monthly for all units. During 2024 and 2025 enforcement of the “scrub in” method was implemented by the burn intensive care unit staff to include all visiting staff as well as the burn staff. The burn unit CLABSI was then placed in a comparison group with the other critical care units within the hospital to show the effectiveness or the ineffectiveness of using the “scrub in” method and the incidents of infection.

**Results:**

It was found that burn ICU practices of “scrubbing in” is effective in preventing CLABSIs. The practice of scrubbing in was presented and encouraged to be practiced in the MICU and hopefully will be implemented as a standard of practice. The staff expressed that this hand hygiene method will help improve patient care and safety.

**Conclusions:**

Over the course of two years of different hand hygiene practices it was found that the use of “scrubbing in” resulted in less CLABSIs.

**Applicability of Research to Practice:**

Hand hygiene is critical in all areas of the hospital. The operating room uses the method of “scrubbing in” to prevent the spread of infectious agents. Our burn unit currently performs daily “scrub in” technique before caring for burn patients. This lead to a correlation found between the practice of “scrubbing in.” This practice significantly lowered the CLABSIs documented when compared to the MICU’s documented CLABSIs. More extensive studies and long-term follow up are required but this work is a valuable steppingstone to identify key practices that can significantly improve our patients care and critical care unit best practices.

**Funding for the study:**

N/A.

